# The expression of thymidine phosphorylase correlates with angiogenesis and the efficacy of chemotherapy using fluorouracil derivatives in advanced gastric carcinoma

**DOI:** 10.1038/sj.bjc.6690719

**Published:** 1999-10

**Authors:** H Saito, S Tsujitani, S Oka, A Kondo, M Ikeguchi, M Maeta, N Kaibara

**Affiliations:** First Department of Surgery, Tottori University School of Medicine, 36-1 Nishi-cho, Yonago 683-8504, Japan

**Keywords:** thymidine phosphorylase, microvessel, gastric cancer, chemotherapy, fluorouracil derivative

## Abstract

The expression of thymidine phosphorylase (TP) and the density of microvessel in advanced gastric carcinoma were examined by immunohistochemistry to evaluate the significance of TP. The expression of TP was negative in 72 cases, positive in 54. The microvessel density correlated with the expression of TP. In total cases, patients with TP-positive tumours survived longer than those with TP-negative tumours. In patients treated with fluorouracil derivatives (FUs), the expression of TP significantly correlated with favourable prognosis and with unfavourable prognosis in those not treated with FUs. The patients with TP-positive tumours, the prognosis of patients treated with FUs was significantly better than that of those not treated with FUs. In patients with TP-positive tumours, treatment with FUs and lymph node metastasis were independent prognostic factors according to the Cox proportional hazards model. Depth of invasion and lymph node metastasis were independent prognostic factors in patients with TP-negative tumours. The determination of the expression of TP might be useful for predicting the efficacy of post-operative chemotherapy using FUs to prevent recurrence in advanced gastric carcinoma patients who undergo curative gastrectomy. © 1999 Cancer Research Campaign


*British Journal of Cancer* (1999) **81**(3), 484–489
© 1999 Cancer Research Campaign
Article no. bjoc.1999.0719

### The expression of thymidine phosphorylase correlates with angiogenesis and the efficacy of chemotherapy using fluorouracil derivatives in advanced gastric carcinoma

**H Saito, S Tsujitani, S Oka, A Kondo, M Ikeguchi, M Maeta and N Kaibara**

First Department of Surgery, Tottori University School of Medicine, 36-1 Nishi-cho, Yonago 683-8504, Japan

**Summary** The expression of thymidine phosphorylase (TP) and the density of microvessel in advanced gastric carcinoma were examined by immunohistochemistry to evaluate the significance of TP. The expression of TP was negative in 72 cases, positive in 54. The microvessel density correlated with the expression of TP. In total cases, patients with TP-positive tumours survived longer than those with TP-negative tumours. In patients treated with fluorouracil derivatives (FUs), the expression of TP significantly correlated with favourable prognosis and with unfavourable prognosis in those not treated with FUs. The patients with TP-positive tumours, the prognosis of patients treated with FUs was significantly better than that of those not treated with FUs. In patients with TP-positive tumours, treatment with FUs and lymph node metastasis were independent prognostic factors according to the Cox proportional hazards model. Depth of invasion and lymph node metastasis were independent prognostic factors in patients with TP-negative tumours. The determination of the expression of TP might be useful for predicting the efficacy of post-operative chemotherapy using FUs to prevent recurrence in advanced gastric carcinoma patients who undergo curative gastrectomy. © 1999 Cancer Research Campaign

**Keywords:** thymidine phosphorylase; microvessel; gastric cancer; chemotherapy; fluorouracil derivative

The majority of patients at an early stage of gastric carcinoma can be cured by surgery, but more than one-half of the patients at an advanced stage of the disease die of carcinoma recurrence, even when they had undergone curative gastrectomy. Recurrence probably arises from the growth of occult micrometastasis cells that have already become established by the time of surgery. It has been found that identical therapeutic management may lead to different clinical outcomes, even in patients at the same stage of the disease. This might be due to the biological characteristics of the tumour itself or the sensitivity of tumour cells to chemotherapeutic agents.

Post-operative adjuvant chemotherapy using fluorouracil derivatives (FUs), one of the most widely used chemotherapeutic agents for the treatment of gastrointestinal tumours, was routinely performed in advanced gastric carcinoma. However, the efficacy of this treatment is a subject of debate because the sensitivity of carcinoma cells to chemotherapeutic agents varies. Thus, identification of specific indicators for sensitivity of these carcinoma cells to anti-cancer drugs would allow a more effective treatment.

It has been reported recently that thymidine phosphorylase (TP) correlated with unfavourable prognosis of various carcinomas via neoangiogenesis (Maeda et al, 1996; Takebayashi et al, 1996). TP was found to be identical to platelet-derived endothelial cell growth factor (PD-ECGF), which was thought to be an angiogenic factor (Ishikawa et al, 1989; Moghaddam et al, 1992). Moreover, TP was known to be an enzyme which converted both 1-(tetrahydro-2-furanyl)-5-fluorouracil and 5′-deoxy-5-fluorouridine (5′-DFUR) to 5-fluorouracil (5-FU) (Kono et al, 1981, 1983). Thus, we speculated that the expression of TP might correlate with the efficacy of chemotherapy using FUs.

In the current study, the expression of TP and the density of microvessels were examined in advanced gastric carcinoma patients with serosal invasion, who had undergone curative resection. Immunohistochemistry investigation was used to evaluate the correlation between the expression of TP and neoangiogenesis or the efficacy of chemotherapy using FUs.

*Received 15 October 1998*
*Revised 26 February 1999*
*Accepted 10 March 1999*

*Correspondence to:* H Saito

### MATERIALS AND METHODS

### Patients

A total of 126 advanced gastric adenocarcinoma patients with serosal invasion who had undergone curative gastrectomy at our institution from January 1976 to December 1995, were used in this study. The patients' ages ranged from 24 to 91 years (average 62.2); 70 were male and 56 were female. The clinicopathological findings were determined according to the rules set forth by the Japanese Society Committee on Histological Classification of Gastric Cancer (Japanese Research Society for Gastric Cancer, 1995). Criteria for consideration as curative resection were the complete removal of a primary gastric tumour, dissection of regional lymph nodes and no macroscopic tumour being left

**Table 1** Correlation between the treatment of fluorouracil derivatives and clinicopathological features

| Variables | Treatment of fluorouracil derivatives | | |
|---|---|---|---|
| | Treated ($n$ = 91) | Untreated ($n$ = 35) | $P$-value |
| Age (years) | 57.4 ± 12.4 | 70.9 ± 12.5 | $P$ < 0.0001 |
| Gender | | | |
| Male ($n$ = 70) | 49 | 21 | NS |
| Female ($n$ = 56) | 42 | 14 | |
| Tumour location | | | |
| Upper ($n$ = 31) | 23 | 8 | NS |
| Middle ($n$ = 53) | 39 | 14 | |
| Lower ($n$ = 42) | 29 | 13 | |
| Size (cm) | 8.2 ± 3.9 | 8.2 ± 3.9 | NS |
| Histology | | | |
| Well differentiated($n$ = 34) | 21 | 13 | NS |
| Poorly differentiated($n$ = 92) | 70 | 22 | |
| Stage | | | |
| II ($n$ = 67) | 53 | 14 | NS |
| III ($n$ = 47) | 29 | 18 | |
| IV ($n$ = 12) | 9 | 3 | |
| Lymphatic involvement | | | |
| Absent ($n$ = 42) | 34 | 8 | NS |
| Present ($n$ = 84) | 57 | 27 | |
| Vascular involvement | | | |
| Absent ($n$ = 45) | 30 | 15 | NS |
| Present ($n$ = 81) | 61 | 20 | |
| Gastric resection | | | |
| Partial ($n$ = 68) | 49 | 19 | NS |
| Total ($n$ = 58) | 42 | 16 | |
| Expression of TP | | | |
| Positive ($n$ = 71) | 51 | 20 | NS |
| Negative ($n$ = 55) | 40 | 15 | |

NS, not significant.

behind. The patients had no metastasis in the liver, peritoneum, or in distant organs at the time of surgery. No other previous or concomitant primary cancer was present. Patients had not received either chemotherapy or radiation therapy before surgery. All had experienced distal partial gastrectomy, proximal partial gastrectomy, or total gastrectomy with regional lymph node dissection to group 1 (D1), group 2 (D2), group 3 (D3), or group 4 (D4) by curative intent. None of the resection margins were positive for tumours. With regard to adjuvant chemotherapy, tegafur (FT-207, Taiho Co., Japan) (Palmeri et al, 1990) was used in 51 patients and uracil-tegafur (UFT, Taiho Co., Japan) (Maehara et al, 1990) in 40 patients; both were administered for 6 months to a year. Of the patients used in our study, 35 had no chemotherapy with FUs. These patients received either 600–800 mg FT twice, or 200–400 mg UFT two or three times daily orally. As a rule, the patients over 70 years old were not treated with anticancer agents in our hospital. Thus, the mean age was 57.4 in patients treated with FUs and 70.9 in patients not treated with FUs; there was a significant difference ($P$ < 0.0001). However, there was no signficant difference in other clinicopathological factors (Table 1). Mitomycin C (MMC) was used in 79 patients as follows: (i) 14 patients – intraperitoneal administration at a dose of 8–10 mg after surgical resection; (ii) 58 patients – intravenous administration at a dose of 8–30 mg after surgical resection and/or on the post-operative day; and (iii) 18 patients – continuous hyperthermic peritoneal perfusion (CHPP) with physiological saline that contained 10 $\mu$g ml$^{-1}$ MMC (Hamazoe et al, 1994). Moreover, cisplatin (CDDP) was used in 14 patients as follows: (i) six patients – intraperitoneal administration at a dose of 90–150 mg after surgical resection; (ii) eight patients – CHPP physiologic saline that contained 125–150 mg CDDP. Data concerning patients' outcomes, including overall survival and either presence or absence of metastases were available from all patients.

### Immunohistochemistry

### *Thymidine phosphorylase expression*

Four-micrometre-thick sections were dewaxed in xylene, dehydrated in ethanol and then heated in a microwave oven (700 W) for 10 min to retrieve antigens. Endogenous peroxidase was blocked by incubation of samples in 3% hydrogen peroxide in methanol. After being washed with phosphate-buffered saline (PBS), they were incubated overnight with anti-dThdPase monoclonal antibody at a 1:500 dilution. This antibody was obtained from the Nippon Roche Research Center (Kanagawa, Japan). This antibody was prepared with an antigen consisting of human dThdPase purified from human colon cancer xenograft HCT116. The characterization of this antigen was reported by Nishida et al (1994). Next, they were incubated with Envision$^+$ reagent (Dako Co. Ltd, Denmark) for 30 min at room temperature. Envision$^+$ reagent is peroxidase labelled polymer conjugated to goat anti-mouse immunoglobulins in Tris–HCl buffer containing carrier protein and an antimicrobial agent. The reaction products were visualized with diaminobenzidine as the chromogen and the sections were counterstained with methyl green. Normal mouse immunoglobulin G was used instead of the primary antibodies for negative controls. The degree of monoclonal antibody reactivity with individual tissue sections was considered positive if unequivocal staining of cytoplasm or nuclear compartment was seen in more than 5% of the tumour cells, as previously reported (Takebayashi et al, 1996).

### *Microvessel detection and counting*

A detection procedure for microvessels was performed using anti-CD34 monoclonal antibody (Nichirei Ltd, Tokyo, Japan). Envision$^+$ reagent was applied for immunoreaction. A single microvessel was defined as any brown immunostained endothelial cell separated from adjacent microvessels, tumour cells, and other connective tissue elements. The stained sections were screened at × 100 magnification (× 10 objective lens and × 10 ocular lens) under a light microscope (Vanox-S, Olympus, Tokyo, Japan) to identify the five regions of the section with the highest number of microvessels. The image was visualized on a computer display (Macintosh 7500/100, Apple Computer Inc., Cupertino, CA, USA) through a colour video camera module (XC-003, Sony, Tokyo, Japan) and colour image freezer (AE-6905C, Atto, Tokyo, Japan). Microvessels were counted in these areas at × 200 magnification (× 20 objective lens and × 10 ocular lens), and the average numbers of microvessels were recorded. The visualized area on the display was determined to be 0.075 mm$^2$. Two observers (ST and HS) did the counting and the mean value was used for analysis. Large vessels with thick muscular walls were excluded in the counts. Observation of the lumen was not required for identification of a vessel.

### Statistical analysis

The association of factors was evaluated by the $\chi^2$ test. The significance of differences among means was determined by Student's $t$-test or Mann–Whitney $U$-test. Differences between survival

**Table 2** Correlation between the expression of TP and clinicopathological features

| Variables | Expression of TP | | |
|---|---|---|---|
| | Positive (*n* = 54) | Negative (*n* = 72) | *P*-value |
| Age (years) | 60.7 ± 14.3 | 61.5 ± 13.6 | NS |
| Gender | | | |
| Male (*n* = 70) | 34 | 36 | NS |
| Female (*n* = 56) | 20 | 36 | |
| Tumour location | | | |
| Upper (*n* = 31) | 13 | 18 | NS |
| Middle (*n* = 53) | 24 | 29 | |
| Lower (*n* = 42) | 17 | 25 | |
| Size (cm) | 8.1 ± 3.5 | 8.3 ± 4.2 | NS |
| Histology | | | |
| Well differentiated (*n* = 34) | 15 | 19 | NS |
| Poorly differentiated (*n* = 92) | 39 | 53 | |
| Lymph node metastasis | | | |
| Absent (*n* = 70) | 32 | 38 | NS |
| Present (*n* = 56) | 22 | 34 | |
| Lymphatic involvement | | | |
| Absent (*n* = 42) | 16 | 26 | NS |
| Present (*n* = 84) | 38 | 46 | |
| Vascular involvement | | | |
| Absent (*n* = 45) | 18 | 27 | NS |
| Present (*n* = 81) | 36 | 45 | |
| Gastric resection | | | |
| Partial (*n* = 68) | 29 | 39 | NS |
| Total (*n* = 58) | 25 | 33 | |
| Lymph node dissection | | | |
| D1 and D2 (*n* = 57) | 20 | 37 | NS |
| D3 and D4 (*n* = 69) | 34 | 35 | |
| Treatment of fluorouracil derivatives | | | |
| Treated (*n* = 91) | 40 | 51 | NS |
| Untreated (*n* = 35) | 14 | 21 | |

NS, not significant

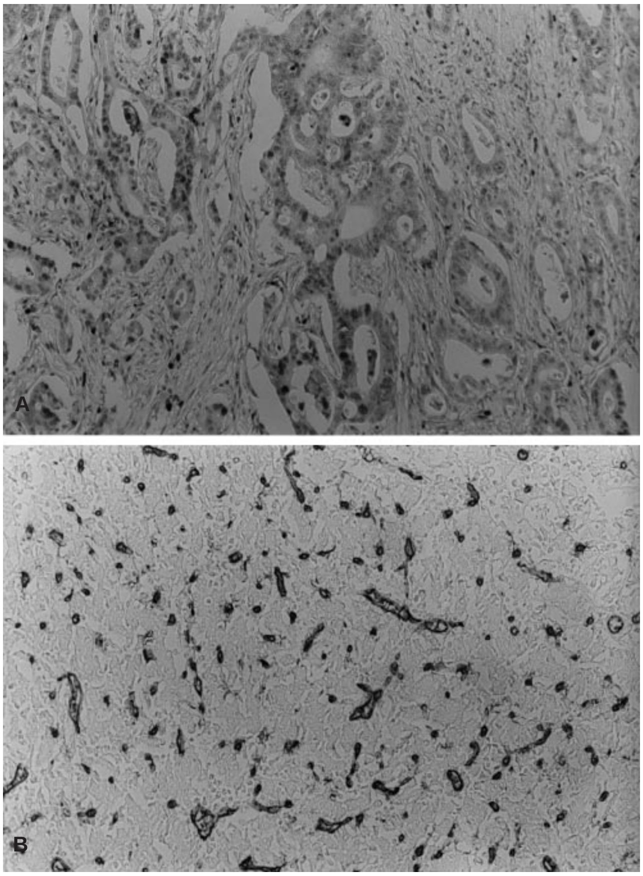

**Figure 1** Typical results of immunohistochemistry. (**A**) TP immunoreactivity was mainly identified in the cytoplasm or nuclear compartments of the carcinoma cells and infiltrating cells (magnification × 500)
(**B**) Immunostaining for anti-CD34 antibody (magnification × 500)

curves were examined with the generalized Wilcoxon test. The influence of each variable on survival was assessed by the Cox proportional hazards model and a step-wise procedure. The accepted level of significance was $P < 0.05$. A Macintosh personal computer system (Stat View software; Abacus Concepts, Inc., Berkeley, CA, USA) was used for all statistical analyses.

### RESULTS

Normal gastric mucosa was not immunoreactive with anti-dThdPase monoclonal antibody. TP was distributed in the cytoplasm or nuclear compartments of the carcinoma cells and infiltrating cells (Figure 1). The expression of TP was negative in 72, positive in 54. There was no correlation between the expression of TP and clinicopathological factors (Table 2). the microvessel density was 49.0 ± 22.7 in TP-positive tumours and 41.5 ± 15.3 in TP-negative tumours. The expression of TP significantly correlated with microvessel density.

The expression of TP significantly correlated with favourable prognosis in total cases (Figure 2). With regard to the treatment by FUs, there was no significant difference in prognosis between the patients treated by FUs and those not treated by FUs in total cases (data not shown). In patients with TP-positive tumours, however, the prognosis of patients given FUs was significantly better than that of those not given FUs ($P < 0.05$), whereas this difference was not observed in patients with TP-negative tumours (Figure 3). The expression of TP significantly correlated with favourable prognosis in patients treated by FUs, and with unfavourable prognosis in patients not treated by FUs (Figure 4).

Multivariate analysis by Cox proportional hazards model and a step-wise procedure was performed to determine the significance of the treatment using FUs based on the status of the expression of TP. Covariates were: age, gender, tumour size, histology, depth of invasion, lymph node metastasis, lymphatic vessel invasion, blood vessel invasion, lymph node dissection, treatment by FUs, treatment by MMC, treatment by CDDP and treatment by CHPP. Treatment by FUs and lymph node metastasis were independent prognostic factors in the patients with TP-positive tumours using Cox proportional hazards model. Depth of invasion and lymphatic vessel invasion were independent prognostic factors in patients with TP-negative tumours (Table 3). Moreover, multivariate analyses were performed based on the treatment by FUs. The expression of TP and lymph node metastasis were independent prognostic factors in the patients given FUs, and only lymph node metastasis was an independent prognostic factor in the patients not given FUs (Table 4).

### DISCUSSION

Solid tumours require neovascularization for growth and metastasis (Folkman, 1990). It is also thought that the degree of tumour

 © 1999 Cancer Research Campaign

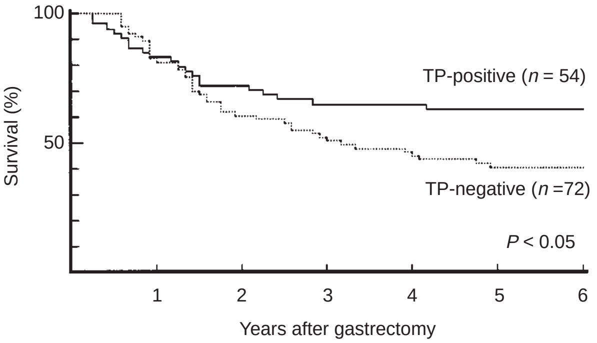

**Figure 2** The correlation between prognosis and the expression of thymidine phosphorylase. The prognosis of the patients with the expression of thymidine phosphorylase is better than that of those without the expression of thymidine phosphorylase ($P < 0.05$)

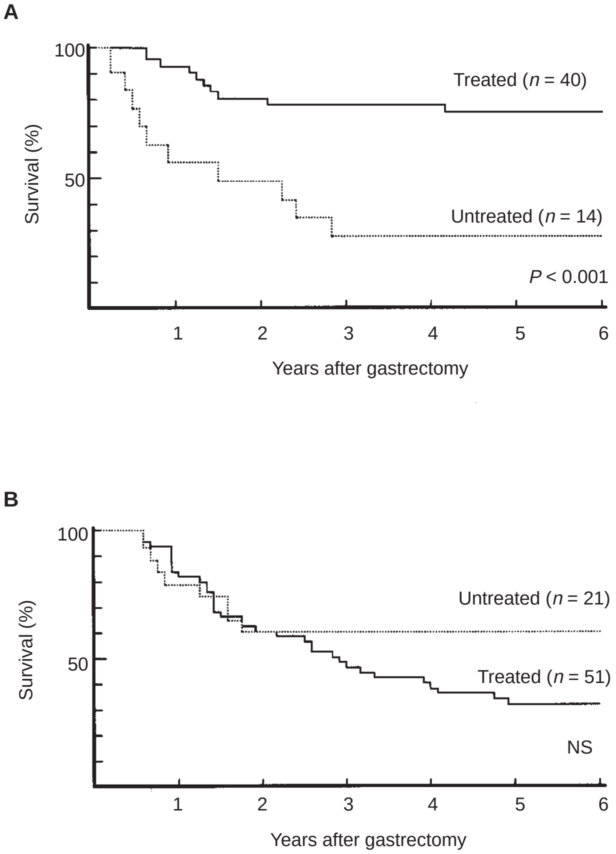

**Figure 3** (**A**) The correlation between the treatment of fluorouracil derivatives and prognosis in patients with the expression of TP. The prognosis of patients treated with fluorouracil derivatives was significantly better than that of the patients untreated with fluorouracil derivatives ($P<0.001$). (**B**) The correlation between the treatment of fluorouracil derivatives and prognosis in patients without the expression of TP. There was no correlation between the treatment of fluorouracil derivatives and prognosis

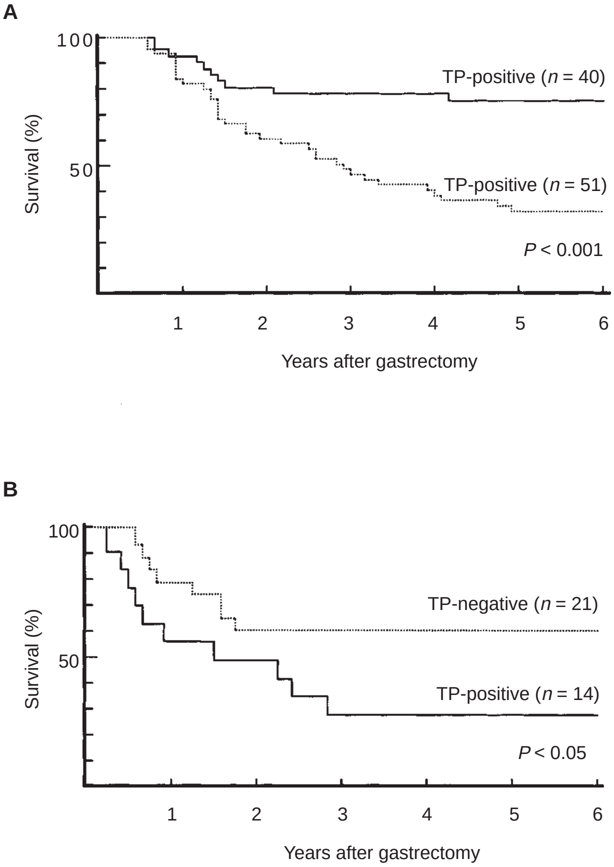

**Figure 4** (**A**) The correlation between the expression of TP and prognosis in patients treated with fluorouracil derivatives. The prognosis of the patients with the expression of thymidine phosphorylase was better than that of the patients without the expression of thymidine phosphorylase ($P < 0.001$). (**B**) The correlation between the expression of TP and prognosis in patients untreated with fluorouracil derivatives. The prognosis of the patients with the expression of thymidine phosphorylase was worse than that of the patients without the expression of thymidine phosphorylase ($P < 0.05$)

angiogenesis is related to clinical outcome; angiogenetic properties are believed to correlate with tumour aggressiveness (Bosari et al, 1992; Weidner et al, 1992; Gasparini et al, 1994; Maeda et al, 1995). Many investigators have demonstrated that tumour cell secretion and activation of various endothelial growth factors, termed angiogenic factors, play crucial roles in the formation of the neovascularization (Kono et al, 1981; Zagzag et al, 1990; Toi et al, 1994). In the current study, we determined the correlation between the expression of TP and the microvessel density in advanced gastric carcinoma. The microvessel density in the TP-positive tumour was significantly higher than that in TP-negative tumour. This result is consistent with a previous report (Maeda et al, 1996).

The prognosis of patients with TP-positive tumours, however, was significantly better than that of patients with TP-negative tumours. Because high microvessel density correlates with poor prognosis in gastric carcinoma (Maeda et al, 1995), as mentioned above, the present findings appear to be rather paradoxical. In fact, Maeda et al reported that the expression of TP correlated with unfavourable prognosis in patients with gastric carcinoma, including the early stage of the disease (Maeda et al, 1996). Thus, we determined the correlation between the expression of TP and post-operative adjuvant chemotherapy using FUs, which were the most widely used chemotherapeutic agents for the treatment of gastric carcinoma in Japan. Both tegafur and uracil-tegafur are 5-FU prodrugs which can be given orally. Recently, it was reported that the expression of TP correlated with the efficacy of 5′-DFUR which was one of the 5-FU prodrugs because TP converted

*© 1999 Cancer Research Campaign*

**Table 3** Association of various factors with overall survival determined by the Cox proportional hazards model and a stepwise procedure in each status of PyNPase expression

| | PyNPase-negative | | PyNPase-positive | |
|---|---|---|---|---|
| **Prognostic factors** | ***P*** | **Hazard ratio** | ***P*** | **Hazard ratio** |
| Depth of invasion (t3 or t4)[a] | 0.0021 | 7.086 | – | – |
| Lymph node metastasis (n0-n3)[b] | – | – | 0.0006 | 2.020 |
| Lymphatic vessel invasion (ly0-1y1)[c] | 0.0093 | 1.374 | – | – |
| Treatment of 5-FU (treated or untreated) | – | – | 0.0251 | 2.993 |

[a]$t_3$, penetrating the serosa; $t_4$, invading adjacent organs. [b]$n_0$, no regional lymph node metastasis; $n_1$, $n_2$ and $n_3$, metastasis in groups 1, 2 and 3 lymph nodes respectively. [c]Lymphatic invasion: 1y0-1y3, grade of lymphatic vessel invasion.

**Table 4** Association of various factors with overall survival determined by the Cox proportional hazards model and a stepwise procedure in both patients given FUs and those not given FUs

| | Treatment of fluorouracil derivatives | | | |
|---|---|---|---|---|
| | Treated | | Untreated | |
| **Prognostic factors** | ***P*** | **Hazard ratio** | ***P*** | **Hazard ratio** |
| Lymph node metastasis (n0-n3)[a] | 0.0019 | 1.615 | 0.0076 | 1.761 |
| Expression of TP (positive or negative) | 0.0009 | 0.284 | – | – |

[a]$n_0$, no regional lymph node metastasis; $n_1$, $n_2$ and $n_3$, metastasis in groups 1, 2 and 3 lymph nodes respectively.

5′-DFUR to 5-FU (Haraguchi et al, 1993; Ishikawa et al, 1998). TP is also one of the enzymes that converted tegafur and uracil-tegafur to 5-FU (Kono et al, 1981, 1983). These findings imply that the expression of TP might correlate with the efficacy of chemotherapy using tegafur and uracil-tegafur.

There was no significant difference in prognosis between the patients treated by FUs and those not treated by FUs in total cases. In patients with TP-positive tumours, however, the prognosis of patients treated by FUs was significantly better than that of those not treated by FUs. Moreover, treatment by FUs and lymph node metastasis were independent prognostic factors in the patients with TP-positive tumours using Cox proportional hazards model. In addition, the expression of TP and lymph node metastasis were independent prognostic factors in the patients given FUs, although only lymph node metastasis was an independent prognostic factor in the patients not given FUs. These results indicated that the expression of TP in tumour cells might closely correlate with the efficacy of chemotherapy using FUs. The prognosis of the patients with TP-positive tumours was worse than that of those with TP-negative tumours who were not treated by FUs. Since a larger number of patients were treated by FUs, our results might be different from the results of previous studies. On the other hand, with regard to the efficacy of other adjuvant chemotherapy except FT and UFT (CDDP, MMC and CHPP), the prognosis of patients given MMC was significantly better than that of patients not given MMC (data not shown). However, there were no significant differences in prognosis in the treatment by CDDP and CHPP. Multivariate analysis indicated that these treatments were not independent prognostic factors in both status of the expression of TP.

Chemotherapy is not generally believed to be very effective for the eradication of massive tumours. However, it can be used to advantage against micrometastases invading the lymph node and/or lymph vessels and disseminated in the abdominal cavity after curative resection (Griswold et al, 1975; Schabel et al, 1976; Schabel et al, 1977). Patients included in the current study had undergone curative gastrectomy. Thus, the correlation between the expression of TP and the efficacy of chemotherapy using FUs might be clear. The efficacy of chemotherapy is presumably dependent on the sensitivity of the tumour cells, which in turn is determined partially by the growth rate. Kubota et al (1995) reported that the overall survival rate of the chemo-sensitive group was significantly better than that of the chemo-resistant group according to the blind study in which their chemo-sensitivity was evaluated by histoculture drug response assay (HDRA). If patients with tumours sensitive to FUs can be identified before the initiation of therapy, FU-based treatment could be selected for this group. Such selection ability would spare patients unlikely to respond to the toxicity of anticancer agents and would allow faster progress in new drug development. It has been reported that the expression of thymidine synthetase (TS) correlated with sensitivity to FUs (Lenz et al, 1995; Leichman et al, 1997). The determination of the expression of TP might also be useful for the purpose of treatment selection. The background of patients treated by FUs, however, was different from that of patients not treated by FUs in this retrospective study. Thus, it is necessary for our findings to be confirmed in randomized controlled studies.

In conclusion, we indicated the close relationship between the expression of TP and microvessel density or the efficacy of chemotherapy using FUs. Determination of the intra-tumoural expression of TP may enable clinicians to decide rationally whether FUs are appropriate first line chemotherapy on a patient-by-patient basis.

© 1999 Cancer Research Campaign

### REFERENCES

Bosari S, Lee AKC, Delellis RA, Wiley RD, Heatley GJ and Silverman ML (1992) Microvessel quantitation and prognosis in invasive breast carcinoma. *Hum Pathol* **23**: 755–761

Folkman J (1990) What is the evidence that tumours are angiogenesis dependent? *J Natl Cancer Inst* **82**: 4–6

Gasparini G, Weidner N, Bevilacqua P, Maluta S, Palma PD, Caffo O, Barbareschi M, Boracchi P, Marubini E and Pozza F (1994) Tumour microvessel density, p53 expression, tumour size, and peritumoural lymphatic vessel invasion are relevant prognostic marker in node-negative breast carcinoma. *J Clin Oncol* **12**: 454–466

Griswold DP Jr (1975) The potential for murine tumor models in surgical adjuvant chemotherapy. *Cancer Chemother Rep* **5**: 187–204

Hamazoe R, Maeta M and Kaibara N (1994) Intraperitoneal thermochemotherapy for prevention of peritoneal recurrence of gastric cancer. *Cancer* **73**: 2048–2052

Haraguchi M, Furukawa T, Sumizawa T and Akiyama S (1993) Sensitivity of human KB cells expressing platelet-derived endothelial cell growth factor to pyrimidine antimetabolites. *Cancer Res* **53**: 5680–5682

Ishikawa F, Miyazono K, Hellman U, Drexler H, Wernstedt C, Hagiwara K, Usuki K, Takaku F, Risau W and Heldin CH (1989) Identification of angiogenic activity and the cloning and expression of platelet-derived endothelial cell growth factor. *Nature* **338**: 557–562

Ishikawa T, Sekiguchi F, Fukase Y, Sawada N and Ishitsuka H (1998) Positive correlation between the efficacy of capecitabine and doxifluridine and the ratio of thymidine phosphorylase to dihydropyrimidine dehydrogenase activities in tumors in human cancer xenografts. *Cancer Res* **58**: 685–690

Japanese Research Society for Gastric Cancer (1995) *Japanese Classification of Gastric Carcinoma.* Kanehara, Tokyo

Kono A, Hara Y and Matsushima Y (1981) Enzymatic formation of 5-fluorouracil from 1-(tetrahydro-2-furanyl)-5-fluorouracil (tegafur) in human tumor tissues. *Chem Pharm Bull* **29**: 1486–1488

Kono H, Hara Y, Sugata S, Karube Y, Matsushima Y and Ishitsuka H (1983) Activation of 5′-deoxy-5-fluorouridine by thymidine phosphorylase in human tumor tissues. *Chem Pharm Bull* (Tokyo) **31**: 175–178

Kubota T, Sasano N, Abe O, Nakao I, Kawamura E, Saito T, Endo M, Kimura K, Demura H, Sasano H, Nagura H, Ogawa N and Hoffman RM The Chemosensitivity Study Group for the Histoculture Drug-response Assay (1995) The potential of the histoculture drug response assay to contribute to cancer patient survival. *Clin Cancer Res* **1**: 1537–1543

Leichman CG, Lenz HJ, Leichman L, Danenberg K, Baranda J, Groshen S, Boswell W, Metzger R, Tan M and Danenberg PV (1997) Quantitation of intratumoral thymidylate synthase expression predicts for disseminated colorectal cancer response and resistance to protracted-infusion fluorouracil and weekly leucovorin. *J Clin Oncol* **15**: 3223–3229

Lenz HJ, Leichman CG, Danenberg KD, Danenberg PV, Groshen S, Cohen H, Laine L, Crookes P, Silberman H, Baranda J, Garcia Y, Li J and Leichman L (1995) Thymidylate synthase mRNA level in adenocarcinoma of the stomach: a predictor for primary tumor response and overall survival. *J Clin Oncol* **14**: 176–182

Maeda K, Chung YS, Takatsuka S, Ogawa Y, Sawada T, Yamashita Y, Onoda N, Kato Y, Nitta A, Arimoto Y, Kondo Y and Sowa M (1995) Tumor angiogenesis as a predictor of recurrence in gastric carcinoma. *J Clin Oncol* **13**: 477–481

Maeda K, Chung YS, Ogawa Y, Takatuska S, Kang SM, Ogawa M, Sawada T, Onoda N, Kato Y and Sowa M (1996) Thymidine phosphorylase/platelet-derived endothelial cell growth factor expression associated with hepatic metastasis in gastric carcinoma. *Br J Cancer* **73**: 884–888

Maehara Y, Watanabe A, Kakeji Y, Baba H, Kohnoe S and Sugimachi K (1990) Postoperative prescription of mitomycin C and UFT for patients with stage IV gastric carcinoma. *Am J Surg* **160**: 242–244

Moghaddam A and Bicknell R (1992) Expression of platelet-derived endothelial cell growth factor in *Escherichia coli* and confirmation of its thymidine phosphorylase activity. *Biochemistry* **31**: 12141–12146

Nishida M, Hino A, Mori K, Matsumoto T, Tanaka Y and Ishitsuka H (1994) Cloning of hybridomas producing anti-human thymidine phosphorylase (dThdPase) and establishment ELISA for measuring dThdPase. *J Jpn Soc Cancer Ther* **29**: 1192

Palmeri S, Gebbia V, Russo A, Armata MG, Gebbia N and Rausa L (1990) Oral tegafur in the treatment of gastrointestinal tract cancers: a phase II study. *Br J Cancer* **61**: 475–478

Schabel FM Jr (1976) Concepts for treatment of micrometastases developed in murine systems. *AJR Am J Roentgenol* **126**: 500–511

Schabel FM Jr (1977) Surgical adjuvant chemotherapy of metastatic murine tumors. *Cancer* **40**: 58–68

Takebayashi Y, Akiyama Y, Akiba S, Yamada K, Miyadera K, Sumizawa T, Yamada Y, Murata F and Aikou T (1996) Clinicopathologic and prognostic significance of an angiogenic factor, thymidine phosphorylase, in human colorectal carcinoma. *J Natl Cancer Inst* **88**: 1110–1117

Takebayashi Y, Yamada K, Miyadera K, Sumizawa T, Furukawa T, Kinoshita F, Aoki D, Okumura H, Yamada Y, Akiyama S and Aikou T (1996) The activity and expression of thymidine phosphorylase in human solid tumors. *Eur J Cancer* **32**: 1227–1232

Toi M, Hoshina S, Takayanagi T and Tominaga T (1994) Association of vascular endothelial growth factor expression with tumor angiogenesis and with early relapse in primary breast carcinoma. *Jpn J Cancer Res* **85**: 1045–1049

Weidner N, Folkman J, Pozza F, Bevilacqua P, Allred EN, Moore DH, Meli S and Gasparini G (1992) Tumour angiogenesis: a new significant and independent prognostic indicator in early-stage breast carcinoma. *J Natl Cancer Inst* **84**: 1875–1887

Zagzag D, Miller DC, Sato Y, Rifkin DB and Burstein DE (1990) Immunohistochemical localization of basic fibroblast growth factor in astrocytomas. *Cancer Res* **50**: 7393–7398

*© 1999 Cancer Research Campaign*
